# CITMIC: Comprehensive Estimation of Cell Infiltration in Tumor Microenvironment based on Individualized Intercellular Crosstalk

**DOI:** 10.1002/advs.202408007

**Published:** 2024-11-05

**Authors:** Xilong Zhao, Jiashuo Wu, Jiyin Lai, Bingyue Pan, Miao Ji, Xiangmei Li, Yalan He, Junwei Han

**Affiliations:** ^1^ College of Bioinformatics Science and Technology Harbin Medical University Harbin 150081 China

**Keywords:** cell‐cell crosstalk, cell infiltration, individualized analysis, network analysis, tumor microenvironment

## Abstract

The tumor microenvironment (TME) cells interact with each other and play a pivotal role in tumor progression and treatment response. A comprehensive characterization of cell and intercellular crosstalk in the TME is essential for understanding tumor biology and developing effective therapies. However, current cell infiltration analysis methods only partially describe the TME's cellular landscape and overlook cell‐cell crosstalk. Here, this approach, CITMIC, can infer the cell infiltration of TME by simultaneously measuring 86 different cell types, constructing an individualized cell‐cell crosstalk network based on functional similarities between cells, and using only gene transcription data. This is a novel approach to estimating the relative cell infiltration levels, which are shown to be superior to the current methods. The TME cell‐based features generated by analyzing melanoma data are effective in predicting prognosis and treatment response. Interestingly, these features are found to be particularly effective in assessing the prognosis of high‐stage patients, and this method is applied to multiple high‐stage adenocarcinomas, where more significant prognostic performance is also observed. In conclusion, CITMIC offers a more comprehensive description of TME cell composition by considering cell‐cell crosstalk, providing an important reference for the discovery of predictive biomarkers and the development of new therapeutic strategies.

## Graphical abstract

CITMIC is a novel method for assessing the extent of cell infiltration in the TME by considering the cell‐cell crosstalk. It can simultaneously infer 86 different cell types and its performance is superior to other methods through comparing cytometry immunophenotyping. The cell infiltration levels can be used to predict cancer patient prognosis, especially in highly staged adenocarcinomas.

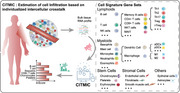

## 1 Introduction

The occurrence and development of tumors are not an isolated event; rather, they develop within a complex microenvironment comprising various components and cell types. This intricate tumor microenvironment consists of surrounding blood vessels, signaling factors, extracellular matrix (ECM), as well as diverse cell types, and these cell types depending on their functional state and developmental origin, include immune cells, stromal cells, and stem cells, in addition to tumor cells. In recent years, there has been increasing attention on the influence of TME on tumors, particularly the impact of immune cells.^[^ ^1^ ^]^ For example, cytotoxic T lymphocytes (CTL) in the tumor microenvironment can inhibit tumor development,^[^ ^2^ ^]^ while dendritic cells (DC) play a role in coordinating anti‐tumor immunity.^[^ ^3^ ^]^ B cells and natural killer (NK) cells have been found to be involved in tumor development.^[^ ^4^ ^]^ Moreover, the role of stromal cells within the cancer microenvironment cannot be ignored. For instance, Cancer‐associated fibroblasts (CAF) are one of the most abundant cell types in the stroma and play multiple roles in cancer progression, including promoting cancer cell growth and proliferation, as well as regulating cancer invasion and immune response.^[^ ^5^ ^]^ Platelets in the tumor microenvironment play a critical role in tumor progression, and targeting systemic antiplatelet therapy is an effective option to improve the efficacy of chemotherapy and immunotherapy.^[^ ^6^ ^]^ In conclusion, the growth, infiltration, and metastasis of tumors are promoted or inhibited through the interaction of non‐malignant and malignant cells within the complex and dynamic TME.^[^ ^7^, ^8^ ^]^ Therefore, delineating the cellular landscape in TME is essential to guide individualized therapy, evaluate prognosis, and develop new therapeutic strategies.

Currently, most studies describing the TME rely on conventional analytical techniques, such as flow cytometry and immunohistochemistry (IHC), which frequently depend on a limited number of preselected marker genes, which restricts the scope of cell types that can be concurrently investigated. The recent rise of single‐cell sequencing data enables transcriptomic analysis of thousands of cells from a single‐cell suspension. However, analyzing large cohorts remains unsuitable and less accurate when examining certain cells, such as rare or cell types that cannot withstand dissociation protocols (e.g., neutrophils), which are often under‐represented.^[^ ^9^ ^]^ Traditional methods are designed for liquid tissues, making it difficult to analyze solid tumors. To overcome these difficulties, a number of in silico tissue dissection tools have emerged in recent years, which can be divided into two main categories: the most common deconvolution approach, CIBERSORT, which estimates the cell components of TME,^[^ ^10^ ^]^ and single sample gene set enrichment analysis (ssGSEA), which uses cell marker gene sets to infer cell abundance.^[^ ^11^ ^]^ However, with the rapid development of single‐cell sequencing technology, many new subtypes of cells in the TME have been identified and distinguished in more detail. Existing tools have significant drawbacks, the main problems are as follows: first, current tools have limitations as they tend to focus on a narrow range of cells, primarily immune cells, and monocytes, neglecting stromal and stem cells that are also present in the TME. xCell is capable of analyzing 64 different cell and molecular components.^[^ ^12^ ^]^ However, with today's cell types being more finely tuned, there is a need for more comprehensive tools to analyze the components of TME. Second, existing algorithms either deconvolution or algorithms based on cell marker gene sets have difficulty distinguishing closely related cells. In particular, when inferring cell abundance using cell‐marker gene sets, each gene feature is considered independently, making it difficult to distinguish closely related cell types. Furthermore, gene‐feature‐based algorithms only provide enrichment scores, which do not allow for comparisons across cell types or provide insight into the abundance of cell types in a mixture. Third, the deconvolution algorithm heavily relies on the structure of the array, which limits its applicability to the specific resource used to develop the array. For instance, CIBERSORT enumerates 22 immune cell types and has shown good estimation results in various Affymetrix microarray studies but has significant limitations when applied to next‐generation sequencing technologies due to its reliance on marker genes, which may be differentially expressed in different samples. CIBERSORT has launched CIBERSORTX to address this issue to some extent.^[^ ^13^ ^]^ However, it requires accompanying single‐cell sequencing data to measure marker genes, which increases the cost of mapping the immune microenvironment.

Furthermore, multicellular life depends on the coordination of cellular activities, which rely on cell‐cell crosstalk in different cell types and tissues of an organism.^[^ ^14^ ^]^ In the TME, the functions between cells are mostly similar and often a physiological activity in which many cells are involved.^[^ ^14^, ^15^ ^]^ For example, in lung adenocarcinoma, there is considerable intercellular communication, particularly between fibroblasts, epithelial cells, myeloid cells, and endothelial cells.^[^ ^16^ ^]^ These cells work together to influence the occurrence and progression of cancer through intercellular communication. Therefore, cell‐cell crosstalk is very common in multicellular life. Starting from the level of cell‐cell crosstalk, this study constructs cell‐cell linkages based on the similarity of inter‐cell functions. By doing so, it infers the cell components present in the TME. This innovative idea can help us better understand the cellular heterogeneity in the TME and improve existing therapeutic approaches.

Here, we developed Cell Infiltration in the Tumor Microenvironment based on Individualized intercellular Crosstalk (CITMIC), which leverages a novel computational approach to comprehensively characterize the TME. Specifically, CITMIC builds individualized cell‐cell crosstalk networks tailored to each sample's transcriptomic background. For each individualized cell network, it captures functional similarities between cells by modeling the connections based on the number of shared biological functions. The TME is then represented by calculating cell‐centric scores within this crosstalk network via network propagation. Our method permits the simultaneous measurement of 86 different cell types, providing an unprecedented breadth of cellular coverage that includes not just immune cells, but also stromal and stem cell populations often overlooked by existing methods. Crucially, the representation of TME by calculating cell centrality scores within the crosstalk network enables the integration of both individual cell types and their functional interactions when assessing the degree of cell infiltration, providing a more comprehensive view than methods considering cells in isolation. To validate our approach, we evaluated RNA‐seq and microarray data from primary cell type samples from different sources to understand the impact of our method on tissue profiling and compared it to cell immunophenotyping methods. We compared our extrapolations to existing methods and demonstrated that CITMIC‐derived scores are more reliable in mixed tissue data analysis. Subsequently, CITMIC was applied to the TCGA‐Skin Cutaneous Melanoma (SKCM) dataset to identify prognostically relevant cell features, which were then utilized to create the cell infiltration risk model to measure patient prognosis. The results indicated a favorable prognostic effect, which was even more pronounced when our score was applied to stage III and IV patients. It was hypothesized that the TME becomes increasingly complex as cancer progresses and that our approach, which begins at the level of cell interactions, would be more effective in high‐stage cancers. This hypothesis was tested by applying it to adenocarcinoma which is known for its rapid metastasis. The results demonstrated that our prognostic model was prognostically effective in high‐stage adenocarcinoma. Additionally, we have also developed an R package which is available on CRAN (https://CRAN.R‐project.org/package=CITMIC).

## 2 Results

### 2.1 Comparison of CITMIC with Previously Published Cell Infiltration Methods

**Figure 1:**
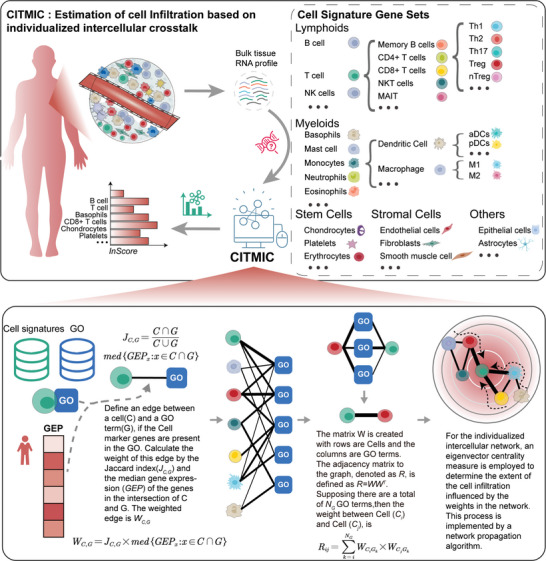
Schematic overview of the CITMIC method.

**Figure 2:**
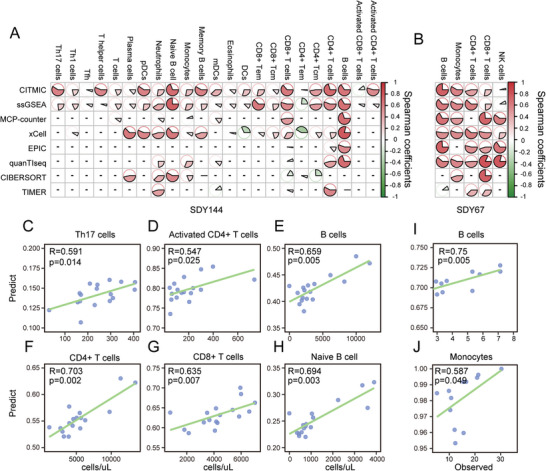
Performance evaluation of CITMIC. A) The correlation coefficient for the comparison of silicon dissection methods and tissue and flow cytometry counts in the whole blood dataset (SDY144); B) The correlation coefficient for the comparison of silicon dissection methods and tissue and flow cytometry counts in the PBMC dataset (SDY67); C–H) Correlation of CyTOF fractions and cell type of Th17 cells, Activated CD4^+^ T cells, B cells, CD4^+^ cells, CD8^+^ cells, and Naive B cells in whole blood dataset (SDY144); I,J) Correlation of CyTOF scores and cell type plots of B cells, Monocytes in PBMC dataset (SDY67). CyTOF, Cytometry by Time‐Of‐Flight.

The CITMIC approach was developed to estimate cell infiltration extent in TME based on individualized cell‐cell crosstalk, and its complete process is shown in **Figure** **1**. To evaluate the effectiveness of CITMIC for tissue dissection, we compared the ability of CITMIC and different methods to infer potential cell enrichment from whole blood and peripheral blood mononuclear cells (PBMCs; available from ImmPort, studies SDY144 and SDY67).^[^ ^17^, ^18^ ^]^ We utilized independent publicly available studies with datasets consisting of large amounts of RNA‐seq data from PBMC (SDY144) or whole blood (SDY67) and the corresponding proportions of immune cell types determined by flow cytometry. These datasets were collected from cancer‐free human donors (see Experimental Section). We calculated an *InScore* (cell Infiltration Scores) for each cell using the expression profiles of these datasets and correlated the *InScores* with the Fluorescence Activated Cell Sorting (FACS) fractions of cell subsets (**Figure** **2A–B**). In SDY144, we identified 23 cell subpopulations and showed a high correlation between *InScore* and FACS scores in cells such as CD4^+^ T cells, Naive B cells, and B cells (Spearman correlation between calculated and actual cell counts *p* value < 0.05; Figure 2C–H). In SDY67, we were able to identify five distinct cell subpopulations. Among these, the correlation coefficient between the *InScore* of inferred B cell and the true cell proportion was as high as 0.75 (Figure 2I). In addition, to evaluate the performance of CITMIC relative to other methods, we compared the performance of CITMIC on these two datasets (SDY67 and SDY144) against other methods (including, deconvolution type methods: CIBERSORT,^[^ ^10^ ^]^ EPIC,^[^ ^19^ ^]^ quanTlseq,^[^ ^20^ ^]^ TIMER,^[^ ^21^ ^]^ and marker gene‐based methods: ssGSEA,^[^ ^11^ ^]^ xCell,^[^ ^12^ ^]^ MCP‐counter^[^ ^22^ ^]^) revealed that the cell types recovered by *InScores* covered all types that could be recovered by other methods. In the comparison with ssGSEA, applying our collection of cell markers genes to ssGSEA in the same number of cell subpopulations estimated, our estimation for various cell subpopulations was more accurate than that (Figure 2A,B). In addition, to validate the stability and reproducibility of our method, we applied our method to two validation sets previously used by xCell (SDY420 and SDY311 see Experimental Section) and validated it with the same treatment of gene expression profiles and FACS scores, which contained 21 cell subpopulations and 165 samples in both datasets. Although in comparison, our approach does not offer a significant performance improvement, our method was able to measure more cell subpopulations such as Th17 cells, Follicular helper T cells (Tfh cells), and T helper cells, and it still correlated significantly with most of them. CITMIC was able to significantly recover 13 and 14 cell subpopulations in SDY311 and SDY420, respectively (Spearman correlation between calculated *InScores* and actual cell counts *p* value < 0.05; Figure S1, Supporting Information).

**Figure 3:**
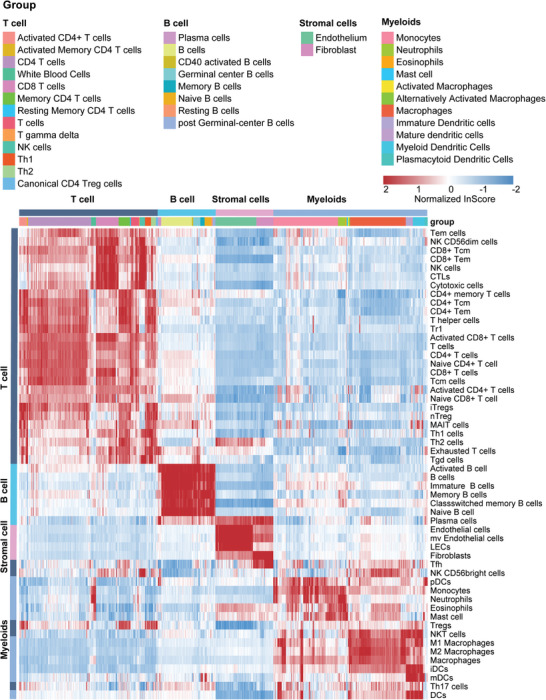
Validate the effectiveness of CITMIC in identifying single‐cell types. Heatmap of the *InScore* profiles calculated from a single‐cell data set (GSE86363) containing 1159 cells. The cells are classified into three categories: myeloid, lymphoid, and stromal cells.

Besides, to validate the effectiveness of my method in identifying single‐cell types, we applied it to a single‐cell gene expression profile (GSE86363) consisting of 1159 cells (containing 34 known cell‐type labels). By calculating the *InScores* for each single‐cell sample and performing cluster analysis, the results showed that the distribution of cell *InScores* was highly consistent with the cell types of original single‐cell samples, suggesting that the *InScore* for each cell population had very high specificity and sensitivity (**Figure** **3**). For example, the myeloid single cell samples including dendritic cells (DCs), mast cells, macrophages, etc. presented high infiltration levels (high *InScores*) for these cells in our method and clustered together. We then explored the biological functions of these cells. Interestingly, amount of key GO processes were found to be shared by the functionally similar cells (Figure S2, Supporting Information). For example, both DCs and M1 macrophages highly express the MHC‐II pathways (GO:0002504 and GO:0019886) and possess strong antigen‐presenting functions.^[^ ^23^, ^24^ ^]^ Similarly, monocytes and macrophages share NF‐kappaB signaling pathways (GO:0007249 and GO:0038061), and which play an important role in the regulation of inflammatory responses.^[^ ^25^ ^]^ These results demonstrated that our method could accurately identify functionally similar cell pairs, which may produce potential crosstalk.

To further validate the applicability of the method, we also applied CITMIC to two other single‐cell gene expression profiling datasets (GSE86357 and GSE86362) and performed clustering analysis. The results showed that the distribution of *InScore* in these datasets was also highly coincident with the cell types (Figures S3 and S4, Supporting Information), which indicates that our method was robust and applicable.

### 2.2 Correlation of Cell *InScore* with Patient Survival in Melanoma

**Figure 4:**
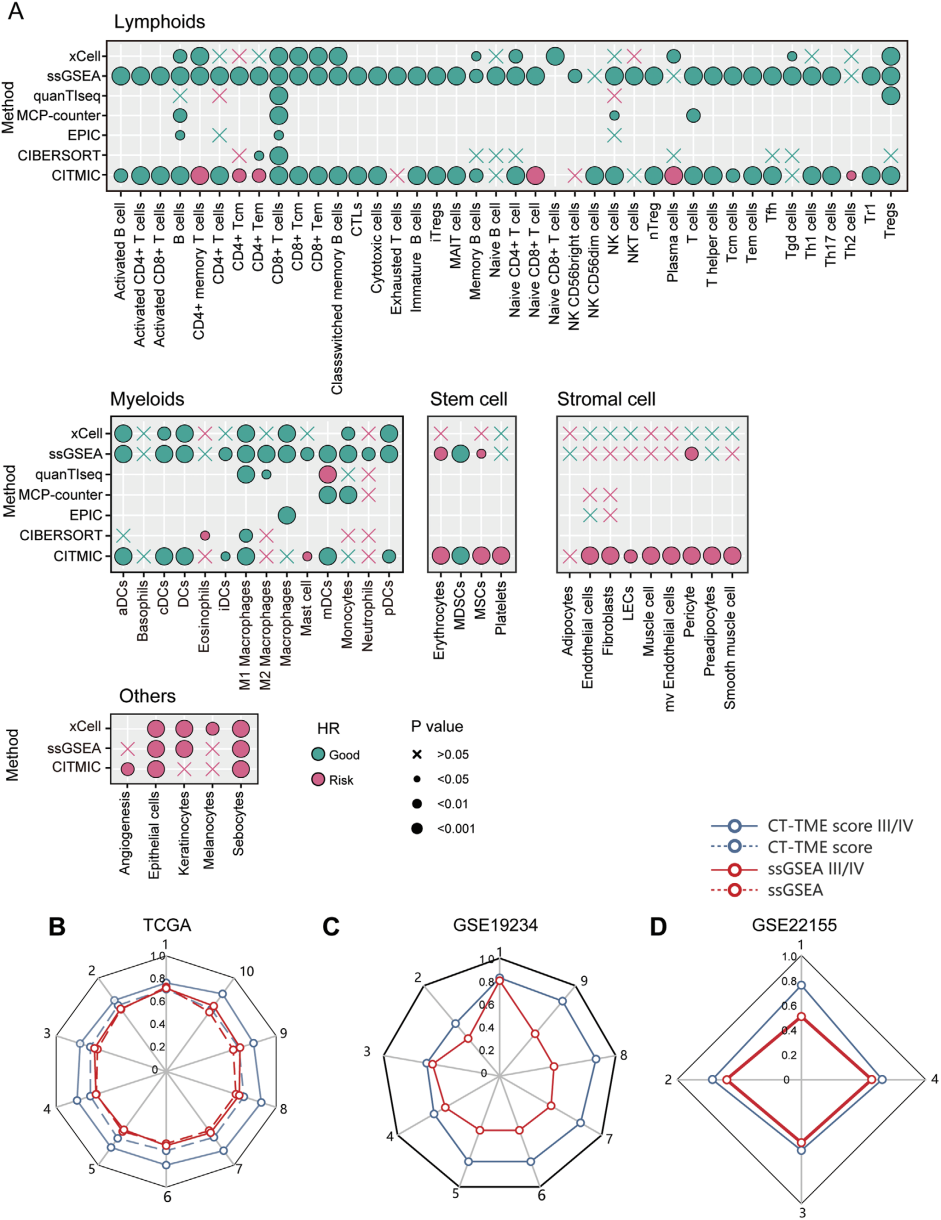
Prognostic ability of the cellular InScore on melanoma patients. A) The cell risk factors are based on single‐factor Cox regression analysis. In this bubble diagram, 71 cells were divided into five groups according to their different cell functions: lymphoid, myeloid, stem cell, stromal cell, and other cells. The color of the bubble indicates whether the cell is a risk or protective factor, with red indicating a risk factor and green indicating a protective factor. The size of the point represents the size of the significance *p*‐value. If the *p*‐value is larger than 0.05, the symbol “X” is employed; B) Comparison of AUROC values over time for 1–10 year overall survival between CITMIC and ssGSEA risk models at TCGA melanoma; C) Comparison of AUROC values over time for 1–9 year overall survival between CITMIC and ssGSEA risk models at GSE19234 melanoma; D) Comparison of AUROC values over time for 1–4 year overall survival between CITMIC and ssGSEA risk models at GSE22155 melanoma.

The TME has a variety of cell subpopulations that play an important role in cancer growth, infiltration, and metastasis. Melanoma is a rare but highly aggressive tumor with increasing incidence.^[^ ^26^ ^]^ Therefore, it is necessary to analyze the TME of melanin to discover new prognostic markers. Here, we applied CITMIC to SKCM to infer the cell *InScores* in the patient's tumor microenvironment. Several cells such as pro‐B cells, hematopoietic stem cells, and common lymphoid progenitor, etc. not present in solid tumor tissues were excluded. To investigate the prognostic effect of cell *InScore* in SKCM, we performed univariate Cox regression analysis of *InScores* for different cell subpopulations to assess the impact of *InScore* on survival. There were 57 cell subpopulations that were significantly associated with prognosis (*p*‐value < 0.05, **Figure** **4**A). Through comparing with other algorithms describing the TME, the *InScore* identified more prognostic relevant cells, of which more were stromal or stem cells, which are considered risk factors (Figure 4A). To verify the plausibility of its prognostic impact, we performed Kaplan‐Meier (K‐M) analysis of the *InScore* of these stem and stromal cells by dividing them into two groups (*InScore*‐high and *InScore*‐low) by median *InScores* and found that patients in *InScore*‐low group have better overall survival (log‐rank *p*‐value < 0.05, Figure S5A, Supporting Information). As our approach focuses more on intercellular relationships, it makes the cell *InScores* not only related to individual cells but also considers their neighboring cells, which can lead to differences in the expression of cell marker genes that are not obvious in different patients, but subpopulations of cells that are involved in multiple biological functions are more likely to show significant differences. Stromal cells are usually the cells that make up the structure of tissues, such as pericyte, endothelial cells, etc., and there is evidence that abnormalities in the pericyte‐endothelial cell signaling network may contribute to tumor angiogenesis and metastasis.^[^ ^27^ ^]^ Immune cells include various types of white blood cells such as T cells, B cells, macrophages, DC cells, etc. These cells perform various immune functions in the immune system, including immune recognition, attacking pathogens, and modulating immune responses.^[^ ^28^ ^]^ Due to their specific functions, immune cells may also exhibit greater heterogeneity in different environments for different immune challenges and tasks. The influence of cellular neighbors in the cell‐cell crosstalk network can be considered by our method to comprehensively measure the changes in cells in different patients. Thus, differences in stromal or stem cells in different patients can be well represented by *InScores* of cells. In addition to this, a large number of studies have proved that stromal cells are involved in tumor development. For example, lymphatic endothelial cells (LECs) promote tumor development as demonstrated in studies,^[^ ^29^ ^]^ preadipocytes are undifferentiated adipocyte precursors that are part of the development and maintenance of adipose tissue, these cells can be transformed into mature adipocytes through differentiation, high‐fat diets may promote malignancy by increasing circulating (bioactive/signaling) lipids which stimulate the growth and migratory activity of cancer cells that do not exhibit a high rate of lipolysis.^[^ ^30^ ^]^ Local defects in adipocytes activated by TME and high circulating concentrations of lipids promote tumor growth in obese patients,^[^ ^30^ ^]^ Vascular endothelial cells are important tissues for maintaining blood perfusion, whereas tumor‐associated endothelial cells interact with tumor cells to form neo‐vessels, thereby supporting tumor progression, promoting metastasis, and participating in the process of resistance to antitumor therapy.^[^ ^31^ ^]^

### 2.3 Construction of *InScore‐*Based Risk Model by Integrating Multiple Cells in Different Stages of Cancer

Given the complexity of the TME, the simultaneous prediction of multiple prognostically relevant cell subpopulations can enhance the accuracy of the clinical prognostic assessment. To this end, we developed a comprehensive cell prognostic risk model (Cell crosstalk‐TME risk model) for the quantification of the tumor microenvironmental status. Specifically, we calculated Cell crosstalk‐TME (CT‐TME) scores for all patients using the TCGA‐SKCM cohort as a training set. From these, 68 cell types were extracted that were significantly associated with the prognosis of TCGA‐melanoma patients (univariate Cox regression analysis, *p*‐value < 0.05). Subsequently, considering the complex interactions between cell types, we further downed the size of cell types significantly associated with prognosis by multivariate Cox regression analysis (*p*‐value < 0.05), obtained 10 cell types significantly associated with prognosis, and constructed a CT‐TME risk model based on their corresponding coefficients, Specifically, the CT‐TME risk model of each sample was as follows: CT‐TME score *=* $\sum_{k=1}^{10}\beta_{k}\times InScore_{k}$. Next, to evaluate the performance of the risk model in terms of patient prognosis, the “surv_cutpoint” (R package “survminer”) function was employed to determine the cutpoint of scores to categorize the patients into the high‐risk and low‐risk groups. The results of the log‐rank test and KM survival analysis indicated that the survival time of patients in the high‐risk group was significantly shorter (Figure S5B,C, Supporting Information, log‐rank *p*‐value < 0.0001). This demonstrates that this risk model is capable of effectively distinguishing patient groups with disparate prognostic risk levels, thereby offering significant value in the assessment of clinical prognosis. Additionally, the receiver operating characteristic curve (ROC) analysis showed that the areas under the ROC curve (AUC) for 1‐, 3‐, 5‐, and 7‐year overall survival were 0.713, 0.69, 0.713, and 0.703, respectively (Figure S5D, Supporting Information). To further validate the advantages of our method in prognostic modeling, we employed the same strategy to establish prognostic models using different cell infiltration methods (quanTlseq, ssGSEA, xCell, EPIC) in the TCGA‐SKCM patient cohort. We then compared the prognostic effects of the different methods based on time‐dependent ROC curves (Figure S5E–H, Supporting Information). The results demonstrated that our CT‐TME risk model significantly outperformed the other methods in prognostic prediction, thereby further substantiating its potential for application in clinical prognostic assessment (Figure S5I, Supporting Information).

**Figure 5:**
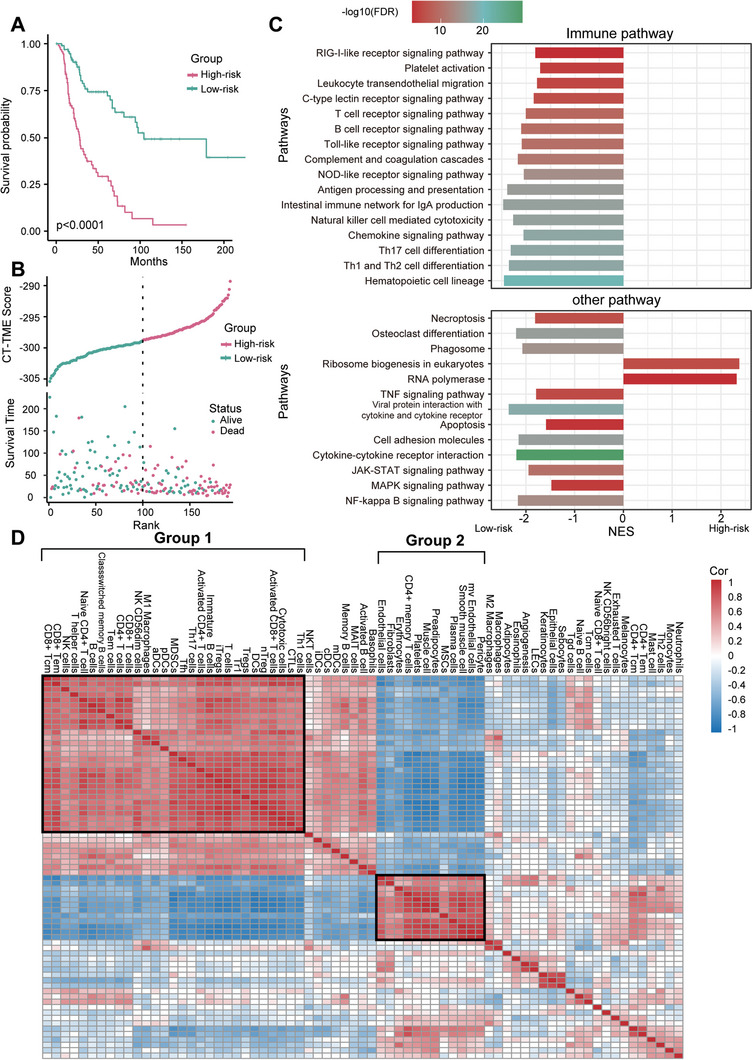
Performance of CT‐TME Risk Model in High‐Stage Melanoma. A) Kaplan–Meier survival curves of patients classified into high‐risk and low‐risk groups using the CT‐TME risk model in high‐stage melanoma; B) Scatter plot depicting risk score and survival time; C) Gene set enrichment analysis of KEGG pathway of differential genes between high‐risk and low‐risk groups; D) Heatmap of cell‐cell correlation coefficients for high‐stage TCGA melanoma patients based on cell *InScores*.

In cancer development, cell‐cell crosstalks are complex, which become more and more frequent and variable as the cancer progresses.^[^ ^32^ ^]^ In our approach, using cell‐cell crosstalk to depict the TME seems to have a better prognostic performance when confronted with more advanced cancers. To verify our assumption, we constructed a CT‐TME risk model by integrating the TCGA‐SKCM cohort of stage III and IV patients as a separate dataset and obtained 15 cell types significantly associated with prognosis (Table S2, Supporting Information), including multiple immune lymphocytes such as CD4^+^ T cells, CD8^+^ T cells and Memory B cells, etc. A substantial body of evidence from numerous studies has demonstrated that immune lymphocytes influence the survival of melanoma patients and regulate the progression of melanoma.^[^ ^33^, ^34^ ^]^ Furthermore, our findings revealed the presence of endothelial cells associated with the prognosis of melanoma, and some studies have proposed that the communication between endothelial cells and melanoma cells exerted an important role in melanoma progression.^[^ ^35^ ^]^ We then chose ssGSEA, a representative method for assessing cell infiltration scores using the cell marker gene sets, as a measure to evaluate the efficacy of our approach in advanced stages. To validate the prognostic performance of the two methods in high‐stage patients, the ROC curve analysis showed that the prognostic performance of our method in stage III and IV patients is significantly higher than applying it to all patients, while the effect is not significantly improved in the ssGSEA method (Figure 4B). To verify the reproducibility of this phenomenon, we acquired two high‐stage melanoma cohorts (GSE22155, GSE19234) in GEO (Gene Expression Omnibus) as validation sets and found the same phenomenon (Figure 4C,D). Moreover, the K‐M survival curves had more significant prognostic results by dividing all patients into the high‐risk and low‐risk groups (**Figure** **5**A,B). Meanwhile, in the two validation sets, using the same thresholds as in the TCGA‐SKCM dataset, patients in the two validation cohorts also showed significant differences in overall survival and decent predictive performance (Figure S6, Supporting Information).

Subsequently, to investigate in depth the biological pathways activated in the high‐risk and low‐risk groups for high‐stage patients, we investigated biological pathways using Gene Set Enrichment Analysis (GSEA) analysis. The results showed that 29 pathways were significantly associated with CT‐TME score (FDR < 0.001), here among them were a large number of immune‐related pathways positively associated with the low‐risk group, such as the “RIG‐I‐like receptor signaling pathway”, “Platelet activation”, “Th17 cell differentiation” and “Th1 and Th2 cell differentiation”(Figure 5C). These pathways might be the factors for the favorable prognosis in the low‐risk group. Among the pathways associated with the high‐risk group, we identified known pathways associated with SKCM risk, such as “ribosome biogenesis in eukaryotes”, where “RNA polymerase” is an attractive therapeutic target for cancer treatment,^[^ ^36^ ^]^ and it is also a player in driving melanogenesis.^[^ ^37^, ^38^ ^]^ Additionally, a recently proposed concept, “ribosomal translational cancers” has emerged.^[^ ^39^ ^]^ To gain a deeper understanding of cells and their crosstalk in the TME, we calculated the correlations between the *InScores* of cells by Spearman correlation analysis and clustered them by Euclidean distance (Figure 5D). Two obvious clusters were observed (Group1 and Group2), where Group1, is dominated by immune cells, including CD8^+^ Tcm, CD8^+^ Tem, Tfh, etc. whereas stem and stromal cells dominated in Group2, which includes stem cells: platelets, Mesenchymal Stem Cells (MSC), and stromal cells: muscle cells, smooth muscle cells, and Pericytes. To gain insight into the functions of these two cell groups, we visualized the Cell‐GO two‐part network in our methodology and looked at the top 500 most relevant BPs in Cell‐GO in both groups, where cells in the Group1 are mainly associated with “cellular process”, “immune system process”, and “response to stimulus”. (Figure S7A, Supporting Information), while the functions related to the Group2 are more complex, including “cellular process”, “metabolic process”, and “response to stimulus”, etc. (Figure S7B, Supporting Information). Thus, Group1 showed more immunological features biologically. Group2, on the other hand, contained a variety of cell types that are biologically involved in support, metabolism, cell development, and growth.

### 2.4 Application of CITMIC in the High‐Stage Adenocarcinomas

**Figure 6:**
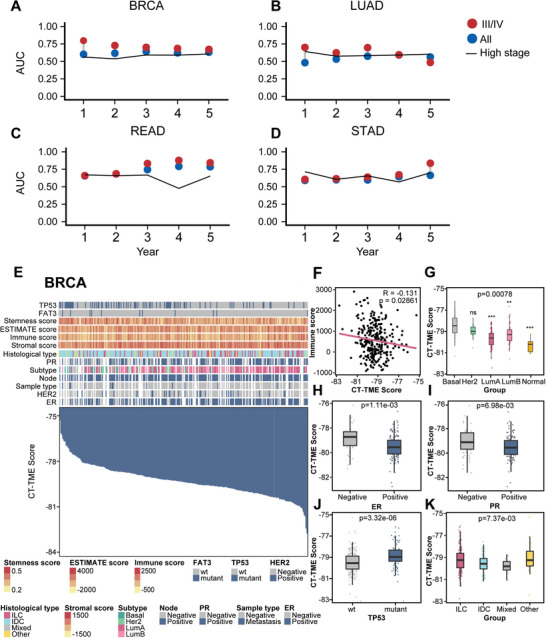
Comparison of the prognostic performance of the CT‐TME risk model in adenocarcinoma between all patients and high‐stage patients, and molecular and clinical features associated with CT‐TME Score in breast cancer. A–D) Comparison of AUROC values over time for 1–5 year overall survival between CITMIC and ssGSEA risk models at high‐stage adenocarcinoma (TCGA‐BRCA, TCGA‐LUAD, TCGA‐READ, TCGA‐STAD). “Highly stage” refers to the ssGSEA risk model for highly staged adenocarcinoma; E) An overview presents an analysis of the association between known molecular and biological processes and CT‐TME Score in high‐stage BRCA. Columns represent patients sorted by CT‐TME Score from low to high (top row). Rows represent molecular and biological processes associated with the CT‐TME Score; F) Correlation of immune score and CT‐TME Score in high‐stage BRCA; G–K) Boxplots of CT‐TME score in high‐stage samples, stratified by molecular and clinical features which include PAM50 subtypes, histological types, status of ER, PR, and mutation of *TP53*. Basal, Basal‐like; Her2, Her2‐enriched; LumA, Luminal A; LumB, Luminal B; Normal, Normal‐like; ILC, invasive lobular carcinoma; IDC, invasive ductal carcinoma; Mixed, mixed Invasive Ductal and Lobular Carcinoma.

The performance of the prognostic model established by our method is decent in high‐stage melanoma patients. We then try to extend the advantages of our method in targeting the prognosis of highly staged melanoma patients to other cancers as a way to confirm the generalizability of our method, i.e., better prognostic ability in the face of different types of high‐stage cancers. Adenocarcinomas are rich in blood vessels, local infiltration and hematogenous metastasis are relatively early, and the cancer progresses rapidly. Moreover, CITMIC is based on cell‐cell crosstalk to depict the TME, and we thought that it would be easier to find differences in adenocarcinomas when applying our method to highly staged patients, so we chose adenocarcinomas to validate our method. We downloaded all adenocarcinoma data from GDC (Genomic Data Commons) TCGA to validate the prognostic performance. Specifically, the difference in prognostic modeling was assessed by comparing the time‐dependent ROC curves between all patients and highly staged patients for BRCA (Breast Invasive Carcinoma), LUAD (Lung Adenocarcinoma), READ (Rectum Adenocarcinoma), and STAD (Stomach Adenocarcinoma) (Materials and methods). It was found that the prognostic models quantified by CITMIC performed better in the face of high‐stage (Stage III and IV) adenocarcinomas (**Figure** **6**A–D). It also validates our conjecture. In addition, further analyses demonstrated that among the significant prognostic cells identified by the respective CT‐TME models in the high‐stage adenocarcinomas, memory B cells emerged as a crucial protective factor in high‐stage LUAD and READ. Interestingly, some evidence was found in the literature for the biological significance of memory B cells in adenocarcinoma.^[^ ^40^ ^]^ Furthermore, in high‐stage breast cancer patients, we identified a range of immune cytokines (including activated B cells, CD8^+^ T cells, mast cells, and NK cells) as significant prognostic factors (Table S3, Supporting Information). These immune cells are integral components of the adaptive immune system and play a pivotal role in immune surveillance and anti‐tumor responses in advanced adenocarcinomas. Thus, the CITMIC method is an effective means of identifying key biomarkers associated with prognosis and treatment response in advanced adenocarcinomas, which is of particular importance in identifying high‐risk subgroups.

**Figure 7:**
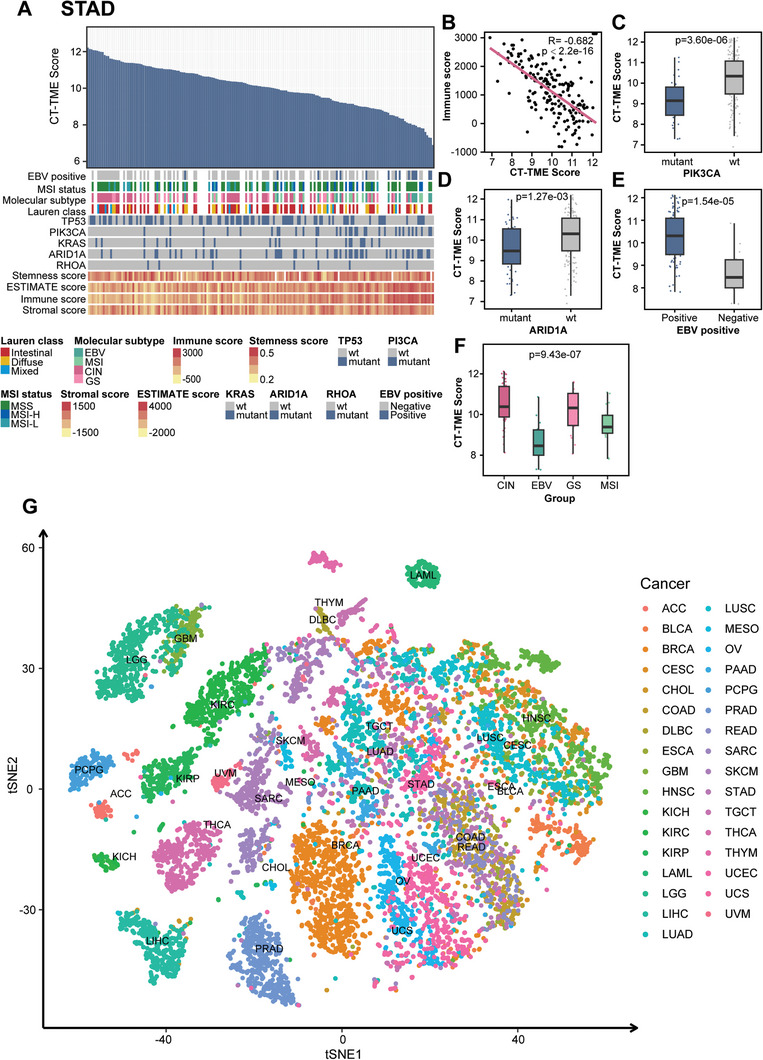
Molecular and clinical features associated with CT‐TME Score in breast cancer, and Portrayal of the tumor microenvironment landscape. A) An overview presents an analysis of the association between known molecular and biological processes and CT‐TME Score in high‐stage STAD; B) Correlation of immune scores and CT‐TME Score in high‐stage STAD; C–F) Boxplots of CT‐TME score in high‐stage samples, stratified by molecular and clinical features which include mutation of *PI3CA* and *ARID1A*, molecular subtype, status of EBV; G) t‐SNE plots of 10328 cancer patients from TCGA (containing 33 cancer types) and targets colored by cancer types. t‐SNE plots were generated using enrichment scores from cell *InScores* for 86 cell types. EBV, Epstein‐Barr virus; MSI, microsatellite instability; GS, genomically stable; CIN, chromosomal instability.

Moreover, we selected BRCA and STAD to investigate whether the CT‐TME score could provide something new insights into adenocarcinoma patients. In BRCA, we found a strong correlation between the CT‐TME score and known clinical, molecular features and estimated scores associated with tumor immunity and stemness^[^ ^40^ ^]^ (Figure 6E). CT‐TME score was highest in the basal subtype patients (Figure 6G), and BRCA patients with high CT‐TME scores were more likely to be estrogen receptor (ER)‐negative and progesterone receptor (PR)‐negative (Figure 6H,I) and enriched for *TP53* mutations (Figure 6J). We noted that invasive lobular carcinoma (ILC) was characterized by a better prognosis than invasive ductal carcinoma (IDC), with a higher CT‐TME score (Figure 6K), proving that our CT‐TME score can be a good assessment of prognosis. We calculated the immunity scores using the R package ESTIMATE^[^ ^41^ ^]^ and found that the CT‐TME score was significantly negatively correlated with the immunity scores, indicating that the CT‐TME score was correlated with immunity (Figure 6F). In STAD, we also tested the correlations between CT‐TME scores and clinical and molecular features of patients (**Figure** **7**A), and found that the CT‐TME scores were significantly negatively correlated with the immune scores (Figure 7B), thereby substantiating the assertion that the CT‐TME scores are related to immunity. Patients with low CT‐TME scores were enriched with *ARID1A* and *PIK3CA* mutations, where *PIK3CA* mutation is associated with a good prognosis in STAD patients.^[^ ^42^ ^]^ *ARID1A* mutation is a biomarker associated with immune response in STAD (Figure 7C,D).^[^ ^43^ ^]^ In addition to this, we also discovered that most of the high CT‐TME scores were EBV‐negative and most of the STAD patients with low CT‐TME scores were Epstein‐Barr Virus (EBV) subtype (Figure 7E,F). Studies have confirmed that EBV‐infected stomach cancer patients have a better prognosis and longer survival.^[^ ^44^ ^]^ These results demonstrate that the CT‐TME score could be a good assessment of the prognosis of cancer patients.

In terms of the TME, different cancers are heterogeneous, and we attempted to use the cell infiltration scores measured by CITMIC as a feature of the TME to differentiate between various cancers. Applying the approach to all cancer patients from GDC TCGA, their CT‐TME scores are calculated and the t‐Distributed Stochastic Neighbor Embedding (t‐SNE) dimensionality reduction technique^[^ ^45^ ^]^ was performed to reduce the dimensionality. It was found that there was some heterogeneity in the cell *InScores* identified by our approach in different cancers (Figure 7G). This concept also highlights the importance of *InScores* for describing the complete cellular heterogeneity of TME for cancer.

## 3 Discussion

In the present study, we introduce CITMIC, a novel computational tool that leverages cell‐cell crosstalk information to comprehensively characterize the cell composition of TME from gene expression data. The term “cell‐cell crosstalk” is not used in the context of direct interactions between cells. Rather, it is used to describe the phenomenon of two cells involved in the same biological process interacting with each other through similar mechanisms (those enriched in the same GO processes), which creates the potential for crosstalk. Considering cell crosstalk will facilitate a more accurate and comprehensive description of the complex tumor ecosystem. It will enable a more precise assessment of the degree of cell infiltration in the tumor microenvironment.

To validate CITMIC's performance, we evaluated its ability to infer cell infiltration across datasets from various independent sources by comparing *InScore* with cytometry immunophenotyping. Next, to demonstrate CITMIC's performance in prognosis, we applied CITMIC to the TCGA‐SKCM dataset. Our analysis revealed that CITMIC outperformed other methods in predicting prognostic markers. We identified several prognostically significant factors, most of which were associated with stromal or stem cells. When assessing the degree of infiltration, CITMIC comprehensively considers cell‐cell crosstalk, thereby improving the accuracy of prognostic prediction.

Subsequently, we developed risk regression models based on cell infiltration scores (*InScores*) that can accurately predict patient clinical outcomes. Considering the changes in TME during cancer development, we also validated the performance of CITMIC in an independent high‐stage TCGA‐SKCM dataset. The results show that CITMIC exhibits a significant improvement in predicting prognostic performance. In addition, we extended this idea to four adenocarcinomas, which are known for their fast tumor metastasis, and obtained satisfactory results. Finally, in our analysis of over 10 000 cases of malignant tumors from GDC TCGA, we found that CITMIC provides an accurate description of cancer heterogeneity using expression profiles from different cancers.

To our knowledge, CITMIC is a powerful tool for analyzing the tumor microenvironment through silico tissue dissection. By incorporating cell‐cell crosstalk, CITMIC offers a more biologically relevant and accurate representation of the tumor ecosystem, thereby opening new avenues for understanding cancer heterogeneity and its clinical implications. This may not only discover new predictive and prognostic markers but also provide important references and guidance for individualized treatment. Currently, CITMIC was developed as an R‐based software, which is available on CRAN (https://CRAN.R‐project.org/package=CITMIC). Future endeavors could be directed toward the integration of multi‐omics data sources and the examination of the potential of CITMIC in guiding targeted immunotherapies or in identifying novel therapeutic targets within the tumor microenvironment.

## 4 Experimental Section

### Cell and Biological Function Signature Gene Sets

To comprehensively characterize the TME, gene signature sets for various cell types were collected from 12 sources, including published cell signature sets (Bindea et al.,^[^ ^46^ ^]^ Charoentong et al.,^[^ ^47^ ^]^ Danaher et al.,^[^ ^48^ ^]^ Davoli et al.,^[^ ^49^ ^]^ He et al.,^[^ ^50^ ^]^ Rooney et al.,^[^ ^51^ ^]^ Tirosh et al.^[^ ^52^ ^]^) and the TME cell estimation methods (MCP‐counter,^[^ ^22^ ^]^ EPIC,^[^ ^19^ ^]^ ImmuCellAI,^[^ ^53^ ^]^ TIDE^[^ ^54^ ^]^ and xCell^[^ ^12^ ^]^). These 12 data sources were merged and obtained 86 cell‐type specific gene signature sets, including 40 lymphocytes, 15 myeloid cells, 11 stem cells, 11 stromal cells, and some other cell types (Table S1, Supporting Information). The combination of multiple sources aimed to provide a comprehensive representation of the cellular heterogeneity within TME.

To construct a patient‐specific Cell‐Cell crosstalk network, the biological processes in Gene Ontology (GO‐BP) were downloaded from the Molecular Signatures Database, MsigDB v6.0.^[^ ^55^ ^]^ Subsequently, biological process gene sets with too few (<15) and too many (>350) genes were excluded, finally, 4171 GO terms were obtained. Incorporating biological process information aimed to capture the functional interactions between different cell types within TME.

### Data Source

*ImmPort data*: This study utilized multiple data sources to evaluate and validate the performance of CITMIC. Independent public studies were downloaded from ImmPort (http://www.immport.org).^[^ ^56^ ^]^ Accession SDY144,^[^ ^17^ ^]^ which contains gene expression data from 17 samples of whole blood, along with Fluorescence activated Cell Sorting (FACS) from 23 different cell types from whole blood samples. Similarly, accession SDY67,^[^ ^18^ ^]^ which contains RNA‐seq counts of 12 pre‐vaccine Peripheral blood mononuclear cell (PBMC) samples, which were transformed into transcripts per million (TPM) and quantile normalized using log2(TPM + 1), and FACS of 5 cell types. In addition, the SDY311 and SDY420 data sets previously used by xCell as validation sets were used for predicting the extent of cell infiltration,^[^ ^57^, ^58^, ^59^, ^60^, ^61^ ^]^ which contained a total of 165 individual whole blood and 18 peripheral blood mononuclear cells for gene expression studies.

*Gene expression data from GEO and TCGA*: To validate the method, the single‐cell gene expression datasets with immune cells and stromal cells were downloaded from the Gene Expression Omnibus (GEO) database with the accession GSE86363 (contains 1159 cells and 34 cell types), GSE86362 (containing 1169 cells and 39 cell types), and GSE86357 (containing 2370 cells and 26 cell types).^[^ ^22^ ^]^ To assess the prognostic utility of CITMIC, survival outcomes (overall survival time and censoring status) and gene expression data were collected from the Genomic Data Commons (GDC) TCGA for various cancer types: 417 patients with stage I‐IV Skin Cutaneous Melanoma, 1069 patients with stage I‐IV breast cancer (BRCA), 347 patients with stage I‐IV stomach cancer (STAD), 496 patients with stage I‐IV Lung Adenocarcinoma (LUAD), and 153 patients with stage I‐IV Rectum Adenocarcinoma (READ). The gene expression data was obtained from the UCSC Xena data portal (http://xenabrwser.net/datapages/).^[^ ^62^ ^]^ For these datasets on the UCSC Xena portal, the gene expression data contain log2‐transformed fragments per kilobase of transcript per million mapped reads (FPKM). To further validate our results, two late melanoma cohorts (accession GSE22155 contains 76 samples and accession GSE19234 contains 44 samples, respectively) were also collected from GEO.^[^ ^63^, ^64^ ^]^ For all datasets, genes expressed in at least 50% of the samples were retained in the gene expression profiles.

### Constructing a Patient‐Specific Cell‐GO Bipartite Network

First, a Cell‐GO bipartite network was constructed based on gene expression activity for each patient. In the bipartite network, the two types of nodes were cells and GO‐BP terms. The weight size of the edges connecting these nodes was measured by the expression activities of the overlapping genes. This means that when a cell and a GO term have overlapping genes, reflecting the involvement of a cell in a particular biological function. Specifically, the weight *W* of an edge is defined as follows:

(1) $$\begin{array}{c} \;W_{C,G}=J_{C,G}\times GEP_{med}\; \end{array}$$

where *J_C,G_* represents the Jaccard coefficient between the cell (C) and GO (G) gene sets, quantifying the overlap between the two gene sets. *GEP_med_* is the median expression of the overlapping genes, calculated as:

(2) $$\begin{array}{c} \;GEP_{med}=med\left\{GEP_{x}:x\in C\cap G\right\}\; \end{array}$$

The Jaccard coefficient between the cell (C) and GO (G) gene sets, reflects the involvement of cell C in biological function G. In addition, considering the differences in the individualized TME, the median expression was calculated, in which the expression activities of the shared genes were used to indicate the infiltration activity of this cell in the individualized TME. This metric provides insight into the expression activities exhibited by these overlapping genes in a given context. Thus, a side weight *W_C,G_* is obtained that is jointly determined by the involvement of cell C in the biological function G and the expression activities of the genes that are shared by C and G.

### Converting the Cell‐GO Network to Cell‐Cell Crosstalk Network

The Cell‐GO bipartite network was then converted to cell‐cell crosstalk network for each patient respectively. Here, the cell‐cell crosstalk refers to the potential interaction between two cells that may engage in analogous mechanisms when involved in the same biological processes.^[^ ^65^ ^]^ In this network, the connection weights between cells will be determined by the GO term shared between them. Specifically, the weights of the edges were defined as follows:

(3) $$\;R_{ij}=\sum_{k=1}^{N_{G}}W_{C_{i},G_{k}}\times W_{C_{j},G_{k}}\;\;$$

For the edge between a cell and a GO in the cell/GO bipartite network, the weight was defined as *W_C,G_*. *N_G_* represents the number of GO terms shared between two cells. The edge weight (*R_ij_*) is the sum of the products of the weights between each cell and the shared GO terms. In other words, a connection between two cells is established only if they share at least one GO term. Moreover, the more GO terms are shared between two cells, and the greater the transcriptional activities of overlapping genes associated with these GO terms, the stronger the connection weight between these two cells. In summary, a patient‐specific cell‐cell crosstalk network was obtained, which was expressed as a cell‐cell adjacency matrix (*R*), where the rows and columns of the matrix represent cells, and the matrix elements represent the weights of the edges between cells. The diagonal elements of this matrix were set to 0 to prevent cells from interacting with themselves.

### Constructing a Cell Infiltration Profiles in a Cancer Patient Cohort

In a cell crosstalk network, the weights of the edges indicate the degree of cell‐cell crosstalk in the patient‐specific tumor microenvironment. It is reasonable to assume that the degree of activity of a cell is influenced by its neighboring cells. Thus, when a cell is connected to more cells with larger edge weights, it is more inclined to exhibit higher vigor in TME. To quantify the extent of cell infiltration, a modified restarted random walk (RWR) algorithm was used to calculate the cell eigenvector centrality score in the weighted network.^[^ ^66^, ^67^ ^]^ Specifically, the modified RWR algorithm is defined as the limiting distribution of a random walk, i.e., the relative frequency at which you would find a random walker at each of the nodes, in the limit of walking for a very long time along the weighted edges. Thus, the starting nodes are not important here, and which are selected from a uniform distribution. What is important is the use of random restarts and edge weights. The greater the centrality of a unit node, the higher the probability that a random walker will traverse that node, resulting in a larger feature vector centrality score. In other words, the greater the centrality of a cell node, the higher the likelihood it will be traversed by the random walker, leading to a larger cell infiltration score (*InScore*).

With this method, the infiltration scores of cells can be computed in each of the transcriptional profile contexts of the sample in a collection cohort by combining the infiltration scores of cells for all samples in the cohort into an *InScore* matrix, with the columns being the samples, the rows are cells, and the elements in the matrix represent the cell *InScores* in the context of a given sample. The cell *InScores* calculated by the RWR algorithm show a power law distribution, and to reduce the influence of extreme values on the results and to improve the stability of the method, log10(*InScore*) was chosen to make the data closer to a normal distribution.

To enable the score to be compared between samples in the cohort, the *InScore* vector of cells for each sample (each column in the *InScore* matrix) was then normalized using the following approach.

(4) $$InScore=\frac{InScore-Min\left(InScore\right)}{Max\left(InScore\right)-Min\left(InScore\right)}\;$$

This normalization step ensures that the cell *InScores* range from 0 to 1 and are comparable across samples in the cohort, allowing for downstream analyses and interpretations.

### Statistical Analysis

The Spearman's rank correlation test was applied to evaluate the correlations between actual and predicted cell infiltration levels. Univariate and multivariate Cox regression analyses were used to identify TME cells associated with patient survival. Kaplan‐Meier survival analysis and log‐rank test were conducted to evaluate the power of prognostic classification based on the TME cells. Continuous variables, such as immune score and CT‐TME score, were compared using Student's t‐test and ANOVA. All statistical tests were two‐sided, with a significance threshold set at *p* < 0.05 unless specified otherwise. All analyses were conducted using R software (version 4.2.1, http://www.R‐project.org).

## Conflict of Interest

The authors declare no conflict of interest.

## Author Contributions

X.Z., J.W., and J.L. contributed equally to this work. X.Z. performed conceptualization, and methodology, wrote the original draft, and software. J.W. performed conceptualization and investigation. J.L. performed data curation, formal analysis, methodology, software, and validation. B.P. performed data curation, methodology, and visualization. X.L. performed methodology, formal analysis, and investigation. Y.H. performed validation, wrote the review, and performed editing. M.J. performed validation. J.H. acquired funding, performed project administration and supervision, wrote the review, and performed editing. All the authors read and agreed to the manuscript.

## Supporting information



Supporting Information

Supplemental Table 1

## Data Availability

CITMIC is freely available on CRAN under the GPL‐v2 license. The source code is available at GiHub (https://github.com/hanjunwei‐lab/CITMIC). All gene expression and clinical data from TCGA GDC cancer patients were accessed through the UCSC Xena platform (https://xenabrowser.net/datapages). Additionally, other gene expression were obtained from the Gene Expression Omnibus (GEO) under the accession numbers GSE86357, GSE86362, GSE86363, GSE22155, and GSE19234. PBMC and whole blood data are available in ImmPort, with data from the following accession numbers:SDY67,SDY144, SDY311, and SDY420. )
