# Supplementary material for: CITMIC: Comprehensive Estimation of Cell Infiltration in Tumor Microenvironment based on Individualized Intercellular Crosstalk
Source: Adv Sci (Weinh). 2024 Nov 5;12(1):2408007. doi: 10.1002/advs.202408007 (PMC11714168; doi:10.1002/advs.202408007)
Supplement: Supplementary file 1 — Supporting Information [file ADVS-12-2408007-s001.pdf]

ADVANCED SCIENCE
Open Access

Supporting Information

for *Adv. Sci.*, DOI 10.1002/advs.202408007

CITMIC: Comprehensive Estimation of Cell Infiltration in Tumor Microenvironment based on Individualized Intercellular Crosstalk

*Xilong Zhao, Jiashuo Wu, Jiyin Lai, Bingyue Pan, Miao Ji, Xiangmei Li, Yalan He and Junwei Han**

Supplementary materials for CITMIC: Comprehensive Estimation of Cellular Infiltration in Tumor Microenvironment based on Individualized Intercellular Crosstalk

Inventory of Supplementary Information

1. Supplementary Note
2. Supplementary Figure S1-7
3. Supplementary Table S1-3

Supplementary Note

Signature gene sets for cells and biological functions

To comprehensively characterize the tumor microenvironment (TME) cell, we curated gene sets for various cell types from 12 different sources, including established cell-type signature sets (Bindea et al, Charoentong et al, Danaher et al, Davoli et al, He et al, Rooney et al, Tirosh et al) and TME cell estimation methods (MCPcounter, EPIC, ImmuCellAI, TIDE, and xCell). The cell-specific gene sets obtained from each source were collated, and the union of marker genes was taken across the 12 data sources. This process resulted in 86 distinct cell type-specific gene signature sets, covering a broad spectrum of immune and non-immune cells: 40 lymphoid cells, 15 myeloid cells, 11 stem cells, 11 stromal cells, and various other cell types (Table S1).
To capture potential functional crosstalk between different cell types in the TME, we downloaded Gene Ontology (GO) biological processes (GO-BP) from the Molecular Signatures Database (MsigDB v6.0). Gene sets representing biological processes were filtered to remove those with fewer than 15 genes or more than 350 genes, refining the set to 4171 GO terms.

Construction of patient-specific Cell-GO bipartite network

For a certain patient, a Cell-GO bipartite network was constructed based on patient gene expression activity and signature gene sets associated with cells and biological functions. In this network, nodes include 86 cell types and 4171 GO-BP terms. The edge weight between a pair of cell and GO-BP nodes was computed as the product of two key factors:

(1) the Jaccard similarity coefficient ($J_{C,G}$) between the gene sets associated with the cell type (C) and the GO-BP term (G);

(2) the median expression value (GEP_{med}) of the genes shared between the cell type and the GO term in the patient's specific transcriptomic profile.

The formula is as follows:

$$W_{C,G} = J_{C,G} \times GEP_{med} \quad (1)$$

We finally obtained an edge weight $W_{C,G}$ that is jointly determined by the involvement of cell C in the biological function G and the expression activities of the genes that are shared by C and G. This method allows us to quantify not only the extent to which a cell type is involved in a given biological function but also how active those shared genes are in the patient's specific tumor microenvironment. According to the above process, a specific Cell-GO bipartite network is constructed for each patient.

Construction of cell-cell crosstalk network

To model the functional crosstalk between cells in the TME, we further convert each Cell-GO bipartite network into a patient-specific cell-cell crosstalk network. In each crosstalk network, nodes represent the 86 cell types, and edge weights represent the strength of functional communication between pairs of cells. Specifically, the weights of the edges were defined as follows:

$$R_{ij} = \sum_{k=1}^{N_G} W_{C_i,G_k} \times W_{C_j,G_k} \quad (2)$$

For the edge between a cell and a GO in the cell/GO bipartite network, we defined the weight as $W_{C,G}$. N_G represents the number of GO terms shared between two cells. The edge weight (R_{ij}) is the sum of the products of the weights between each cell and the shared GO terms. This effectively captures how strongly two cell types share involvement in particular biological functions. Finally, we constructed a specific cell-cell crosstalk network for each patient.

Inference of cell infiltration levels through cell-cell crosstalk network

Within each patient-specific cell-cell crosstalk network, the edge weights represent a quantitative measure of the strength of functional overlap in biological processes between cell types in the TME. Cells may interact through similar mechanisms, leading to potential functional crosstalk.

To quantify the degree of cell infiltration within the patient's TME, we employed the Random Walk with Restart (RWR) algorithm, which is a random walker starting from a specific node, moving along the edges with probabilities proportional to edge weights, simulating the likelihood of the walker visiting other nodes in the network. After each step, the walker has a probability of returning to the starting node, known as the restart probability.

We constructed a probability transition matrix *P* by column-normalizing the adjacency matrix *R*, where:

$$P_{ij} = \frac{R_{ij}}{\sum_{i=1}^{N_C} R_{ij}} \quad (3)$$

Here, P_{ij} represents the probability of transitioning from node *j* ($Cell_j$) to node *i* ($Cell_i$). N_C is the aggregate count of cells.

The process of RWR is as follows:

$$e^{t+1} = (1 - r)Pe^t + re^0 \quad (4)$$

e^0 is the initial probability vector, where all cell nodes are assigned equal probabilities, with the sum of probabilities across all nodes equal to 1. e^t is the probability vector at step t, representing the node probabilities at that step. P stands for the probability transition matrix of the network. r is the restart probability, which denotes the likelihood of a node returning to the initial node during the RWR. The RWR algorithm was implemented using the 'PageRank' function from the 'igraph' package in R. The restart probability (r) was set to 0.9, a value commonly used to ensure a high likelihood of returning to the starting node, thereby emphasizing the local structure of the network around each cell type. This setting ensures that the eigenvector centrality score is strongly influenced by the immediate neighborhood of a cell in the crosstalk network, reflecting its importance and infiltration level within that local context. For each cell type in the crosstalk network, we calculated an eigenvector centrality score, which represents the probability of a random walker visiting that particular cell node. Higher centrality scores indicate that the cell is more likely to be traversed by the random walker, suggesting a higher level of involvement in the overall cellular crosstalk and, by extension, a higher infiltration level (*InScore*) within the patient's TME.

The *InScore* matrix was constructed by aggregating the cell infiltration scores across all patient samples. Given that *InScore*s follow a power-law distribution, we applied a log10 transformation to the data, aiming to approximate normal distribution. This transformation reduces the influence of extreme values and improves the stability of the analysis. Finally, we used a min-max normalization approach, where each sample's infiltration score was rescaled to a range between 0 and 1, ensuring comparability across samples in the cohort and facilitating subsequent analyses.

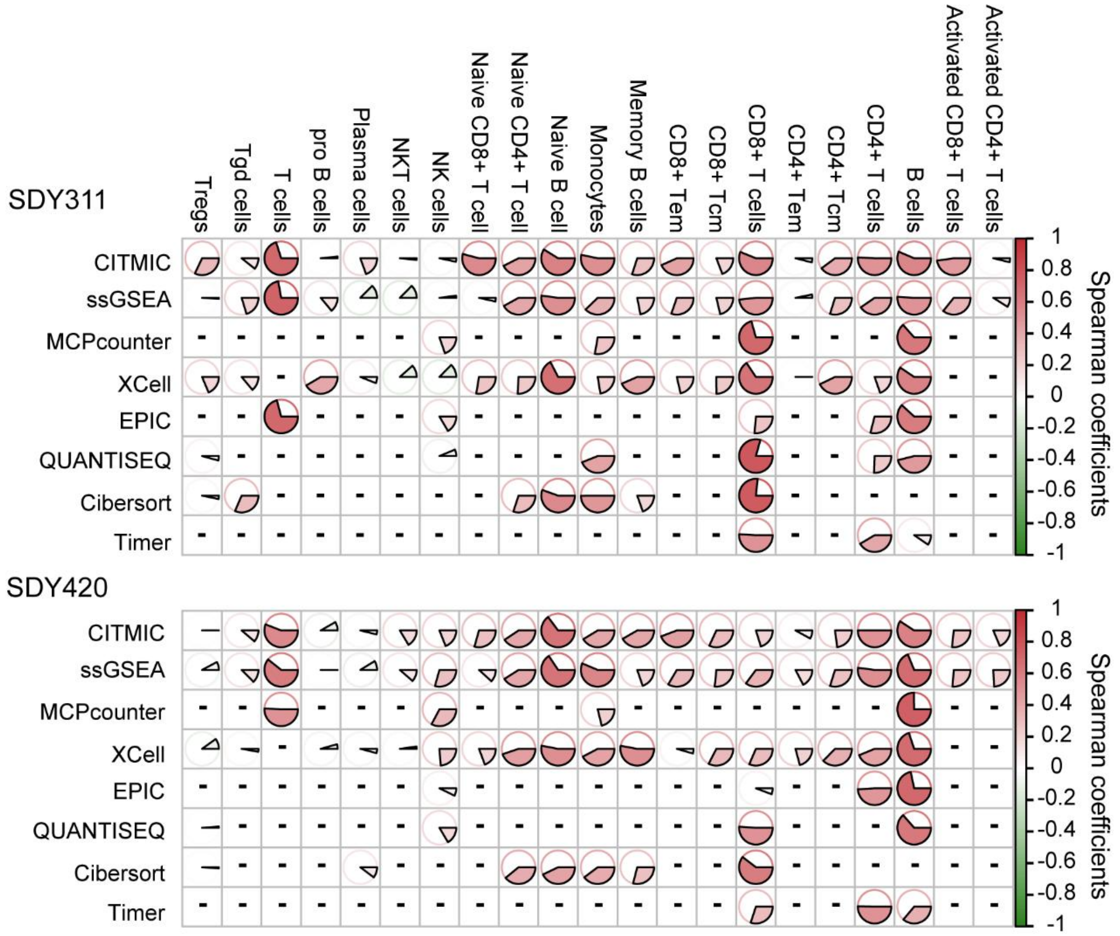

Supplementary Figure S1. The correlation coefficient for the comparison of silicon dissection methods and tissue and flow cytometry counts in the whole blood dataset (SDY311 and SDY420).

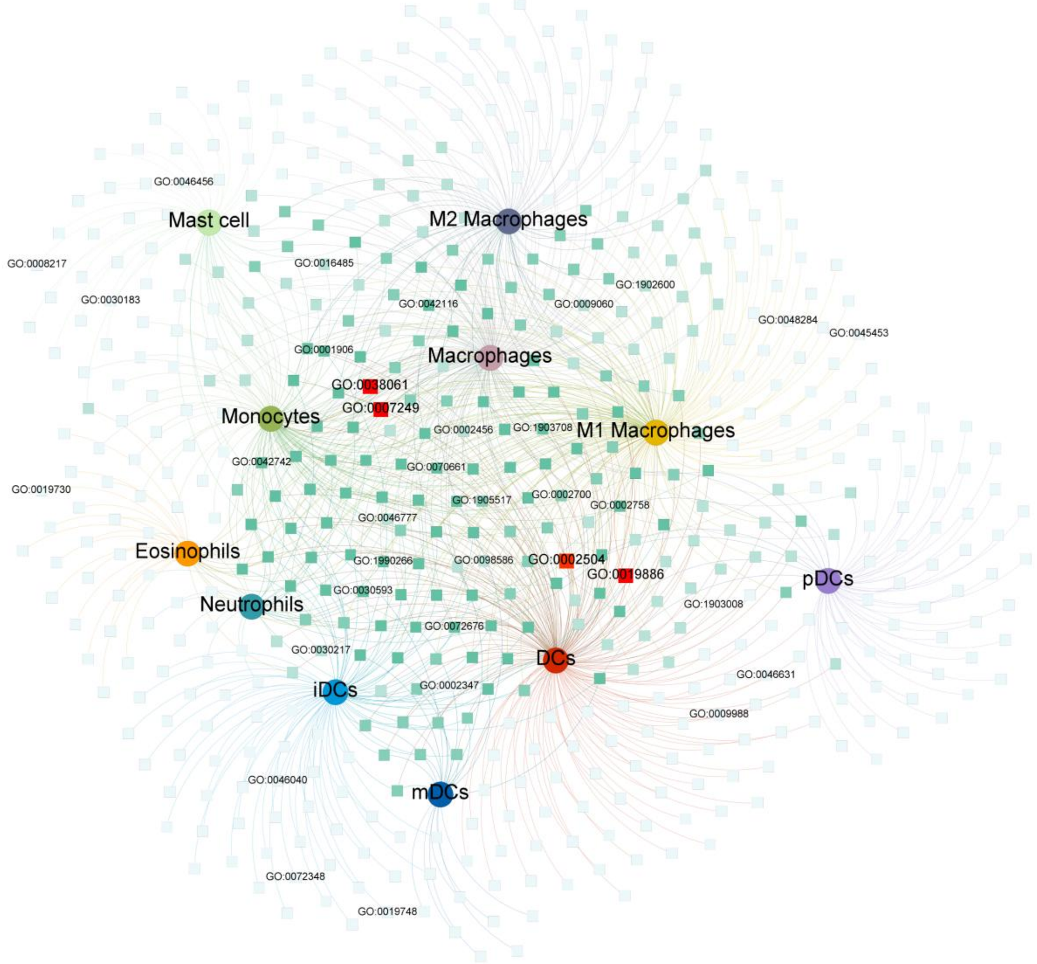

Supplementary Figure S2. The Cell-GO bipartite network in the Cell-GO myeloid cell subpopulation at GSE86363.

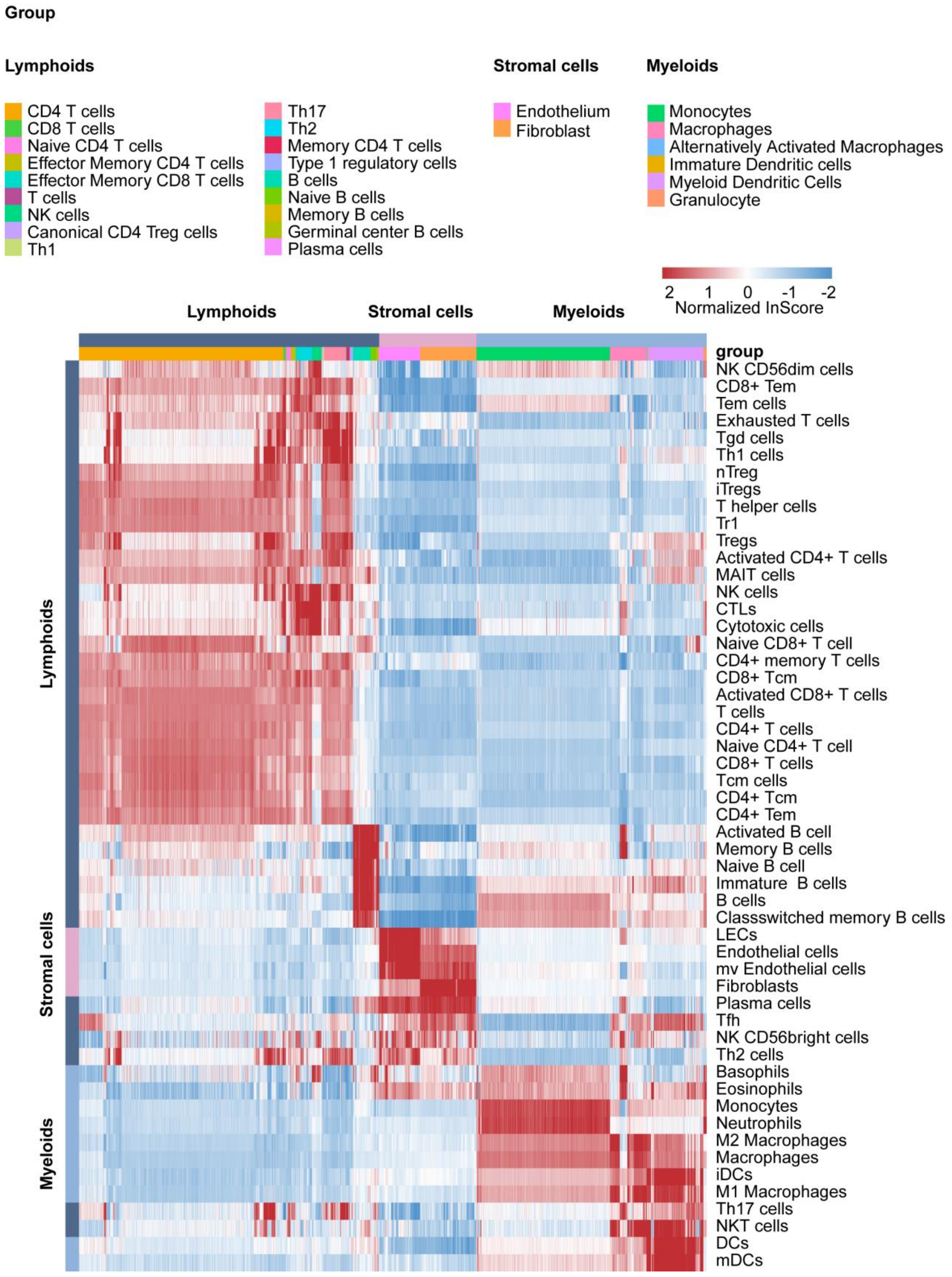

Supplementary Figure S3. Heatmap of the *InScore* profiles calculated from a single-cell data set (GSE86357) containing 2,370 cells. The cells are classified into three categories: myeloid, lymphoid, and stromal cells.

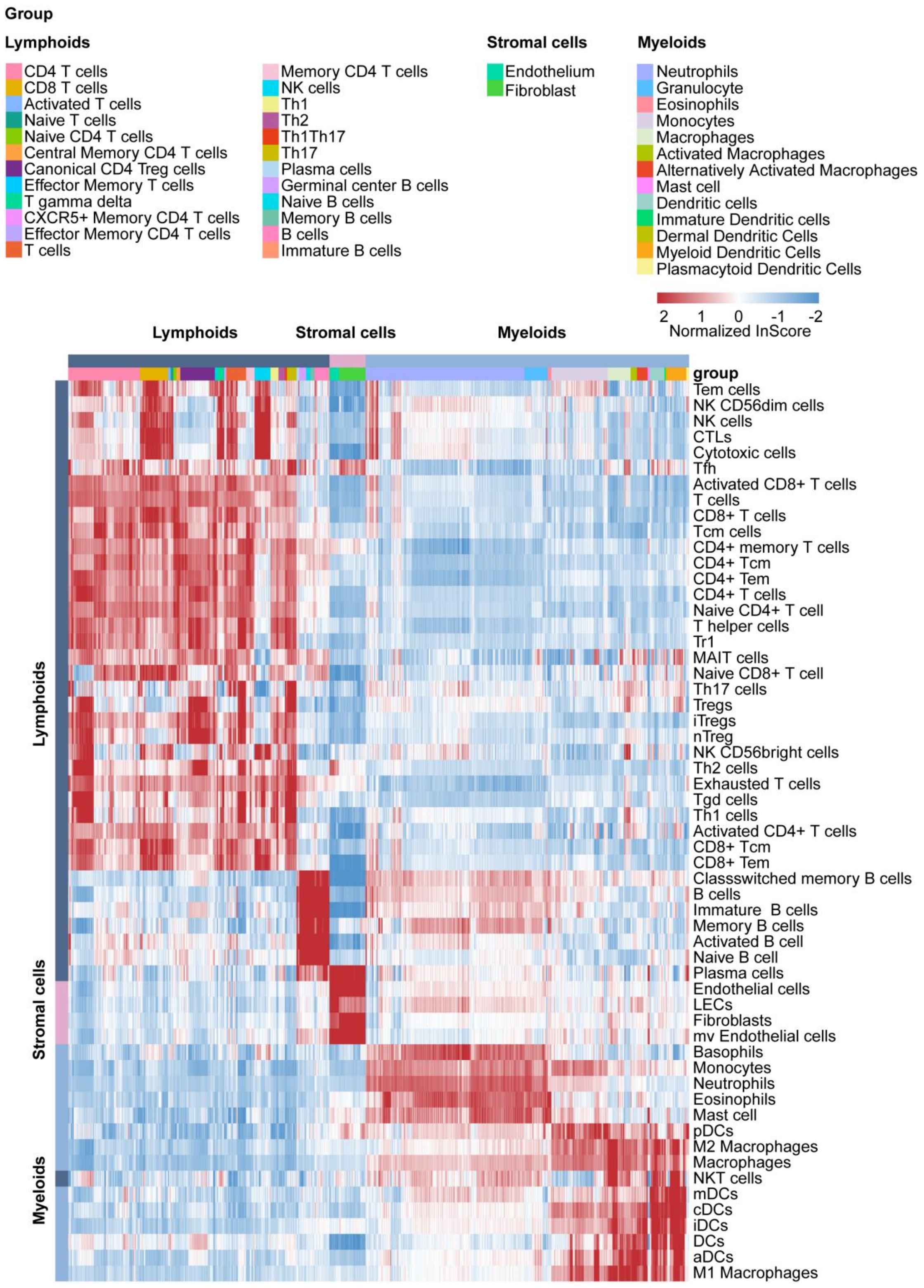

Supplementary Figure S4. Heatmap of the *InScore* profiles calculated from a single-cell data set (GSE86362) containing 1,169 cells. The cells are classified into three categories: myeloid, lymphoid, and stromal cells.

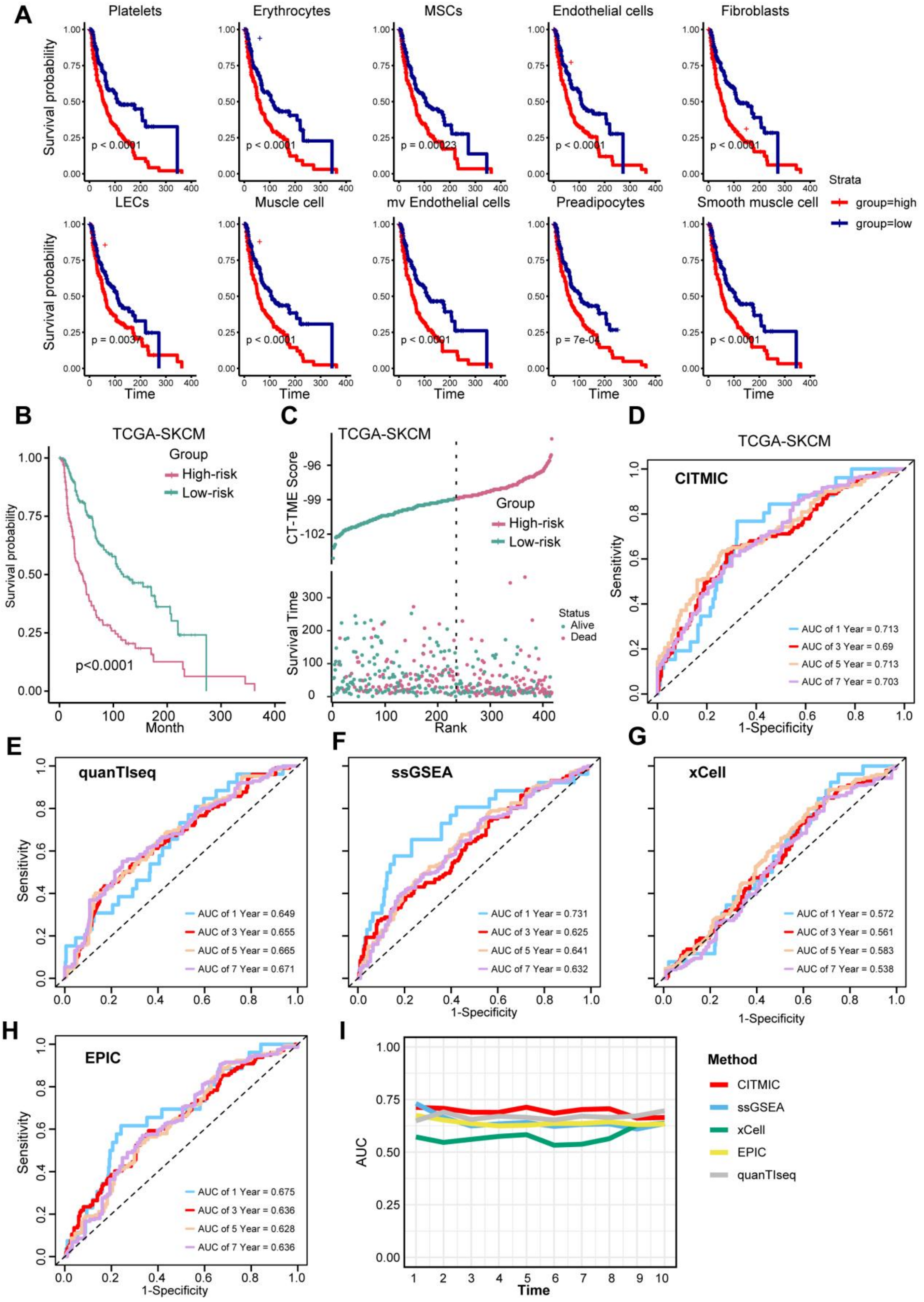

Supplementary Figure S5. (A) Kaplan–Meier survival curves of patients classified into *InScores* of different stemness and stromal cells significantly associated with prognosis in high-stage melanoma. **(**B**)** Kaplan–Meier survival curves of patients

classified into high-risk and low-risk groups using the CT-TME risk model in high-stage melanoma. (C) Scatter plot depicting risk score and survival time. (D) Time-dependent ROC curves for prognosis prediction of the CT-TME risk model for 1-, 3-, 5- and 7-year overall survival in TCGA-SKCM. (E-H) Time-dependent ROC curves for prognosis prediction of risk model based on different methods (quanTIseq, ssGSEA, xCell, EPIC) for 1-, 3-, 5- and 7-year overall survival in TCGA-SKCM. (I) Comparison of AUROC values over time for 1-10 year overall survival between CITMIC and other method risk models at all TCGA melanoma samples.

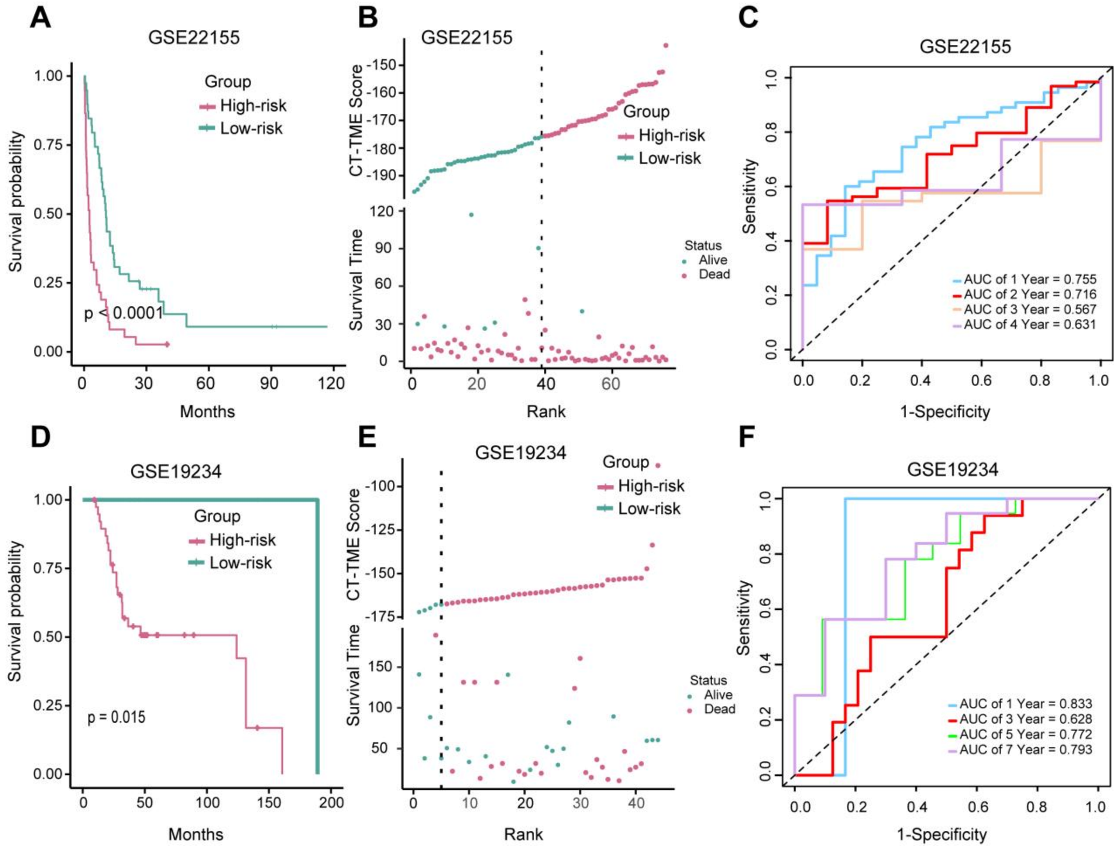

Supplementary Figure S6. (A, D**)** Kaplan–Meier survival curves of patients classified into high-risk and low-risk groups using the CT-TME risk model in GSE22155 and GSE19234. (B, E) Scatter plot depicting risk score and survival time in GSE22155 and GSE19234. (C, F) Time-dependent ROC curves for prognosis prediction of the CT-TME risk model in GSE22155 and GSE19234.

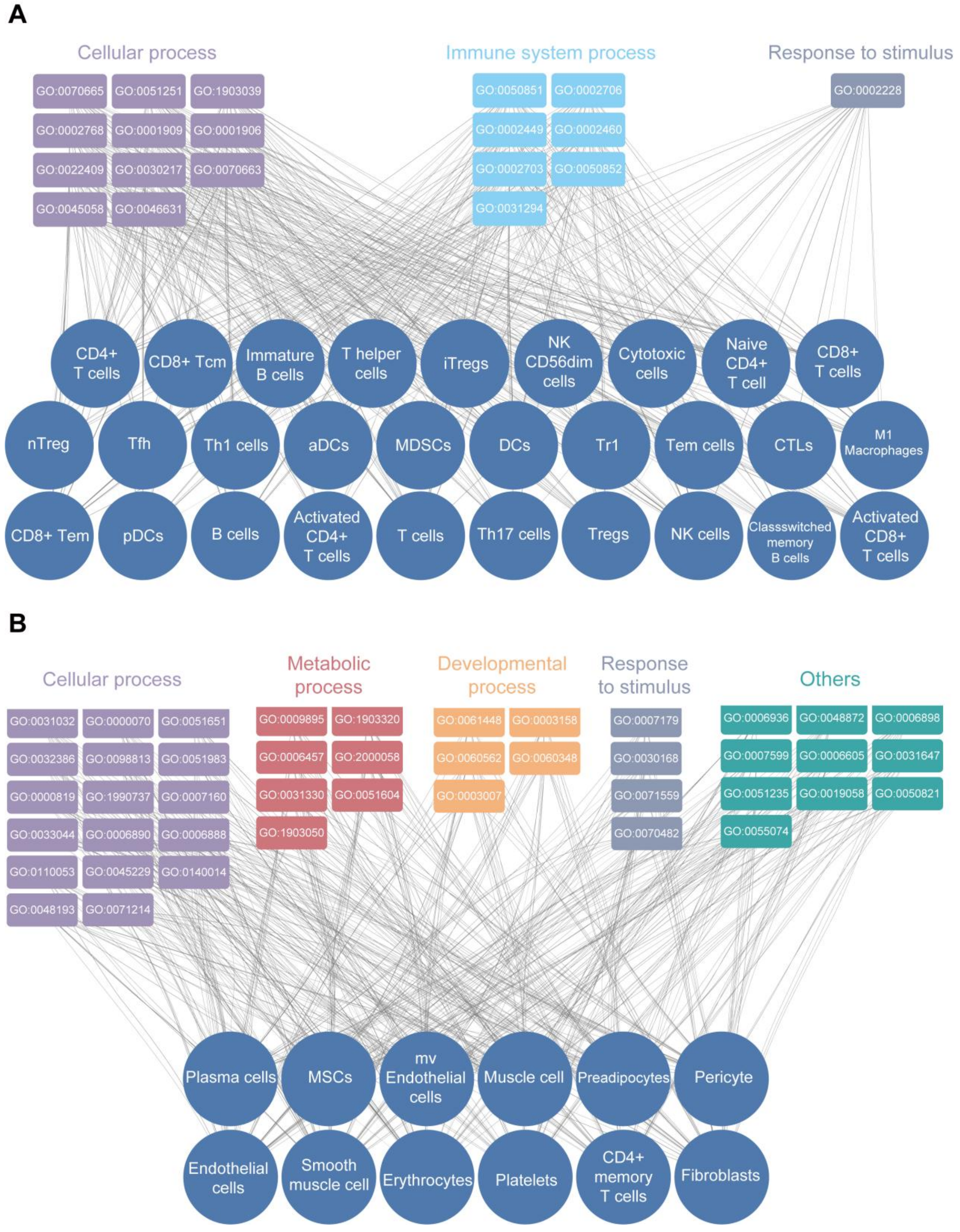

Supplementary Figure S7. (A**)** The Cell-GO network in our methodology contains the top 500 most relevant BPs in Cell-GO in Group1. (B) The Cell-GO network in our methodology contains the top 500 most relevant BPs in Cell-GO in Group2.

Supplementary Table S1. The 86 cell type maker gene sets

(see Supplementary Table S1.xlsx)

Supplementary Table S2. Significant cells identified by multivariate Cox regression analysis associated with overall survival in the high-stage TCGA-SKCM dataset.

Cell	HR	HR.95L	HR.95H	P-value
Activated CD8+ T cells	1.61E+12	1.79E+03	1.44E+21	7.53E-03
CD4+ T cells	1.08E-36	9.94E-62	1.17E-11	4.87E-03
CD8+ T cells	4.79E-30	3.29E-52	6.96E-08	9.52E-03
CD8+ Tcm	2.46E-18	4.22E-33	1.43E-03	1.94E-02
CD8+ Tem	3.80E-25	4.93E-48	2.93E-02	3.65E-02
Endothelial cells	7.44E-34	1.25E-52	4.43E-15	5.43E-04
iTregs	2.09E+15	5.14E+05	8.50E+24	1.78E-03
M1 Macrophages	1.59E-35	2.45E-58	1.03E-12	2.79E-03
mDCs	1.28E-05	3.36E-10	4.88E-01	3.63E-02
Memory B cells	4.53E-15	2.47E-24	8.31E-06	2.41E-03
MPP	1.05E+21	1.35E+00	8.25E+41	4.86E-02
Naive CD8+ T cell	3.49E+22	3.05E+07	3.98E+37	3.34E-03
NK CD56dim cells	2.93E-14	9.85E-25	8.71E-04	1.13E-02
nTreg	1.72E-14	1.14E-23	2.59E-05	3.29E-03
Th1 cells	2.53E-17	1.93E-29	3.31E-05	7.26E-03

Supplementary Table S3. Significant cells identified by multivariate Cox regression analysis associated with overall survival in the high-stage adenocarcinoma datasets respectively (TCGA-BRCA, TCGA-LUAD, TCGA-READ, and TCGA-STAD).

Cancer type	Cell	HR	HR.95L	HR.95H	P-value
BRCA	Activated B cell	3.77E-03	4.86E-05	2.93E-01	1.20E-02
BRCA	CD8+ Tcm	1.65E-12	9.60E-24	2.82E-01	3.98E-02
BRCA	Mast cell	1.10E-08	1.71E-16	7.09E-01	4.58E-02
BRCA	NK cells	2.72E-16	3.44E-27	2.15E-05	5.12E-03
BRCA	Smooth muscle cell	1.21E-10	1.47E-20	9.99E-01	5.00E-02
LUAD	Memory B cells	3.73E-09	2.14E-15	6.51E-03	8.13E-03
READ	Memory B cells	6.37E-19	1.03E-32	3.95E-05	9.72E-03
STAD	Hepatocytes	2.07E+02	3.92E+00	1.09E+04	8.40E-03
STAD	iTregs	5.70E-06	1.39E-09	2.34E-02	4.45E-03
STAD	NKT cells	1.14E+07	1.66E+01	7.89E+12	1.78E-02
